# Supplementary material for: Polynucleotides Suppress Inflammation and Stimulate Matrix Synthesis in an In Vitro Cell-Based Osteoarthritis Model
Source: Int J Mol Sci. 2023 Jul 31;24(15):12282. doi: 10.3390/ijms241512282 (PMC10418450; doi:10.3390/ijms241512282)
Supplement: Supplementary file 1 [file ijms-24-12282-s001.zip › ijms-2461478-supplementary.pdf]

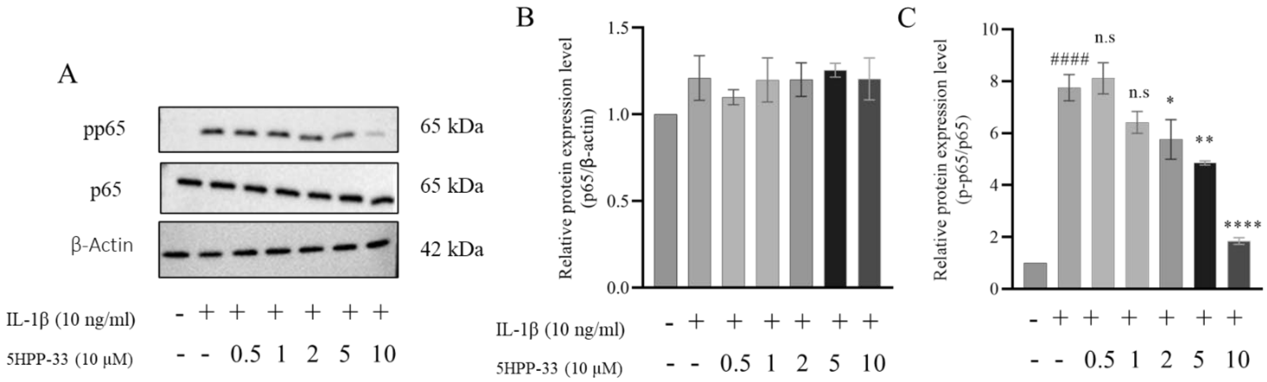

Supplementary Figure S1: Concentration dependent effect of 5HPP-33 inhibitor on pp65 and p65 protein expression levels. (A) Western blot analysis depicting the expression of pp65 and p65 proteins in response to different concentrations of the inhibitor, 5HPP-33. (B, C) Quantitative analysis of the protein expression levels, presented as mean ± standard deviation (n = 3). Statistical significance is denoted as n.s (no significance), ####p < 0.0001 compared to the control group, *p< 0.05, **p < 0.01, ***p < 0.001, and ****p < 0.0001 compared to the IL-1β-treated group.
